# A Molecular and Structural Perspective on Bluetongue Virus Entry and Assembly

**DOI:** 10.3390/pathogens15050470

**Published:** 2026-04-27

**Authors:** Polly Roy

**Affiliations:** 1 Institute for Translational Virology and Innovation, Department of Internal Medicine, Morsani College of Medicine, University of South Florida, Tampa, FL 33612, USA; proy15@usf.edu or roy@uab.edu; 2 The Global Virus Network, Tampa, FL 33612, USA

**Keywords:** bluetongue virus, structure, viral entry, RNA packaging, LLPs

## Abstract

Bluetongue virus (BTV), the prototype of the genus Orbivirus, infects livestock, causing high morbidity and mortality and impacting global trade. BTV is a non-enveloped, double-capsid virus, composed of seven structural proteins and a genome of 10 double-stranded RNA segments. This manuscript highlights our group’s recent findings on the molecular and structural mechanisms underlying BTV entry and assembly during replication. Viral entry is a stepwise, pH-dependent process. The outermost protein, VP2, attaches to sialic acids and senses the acidic pH of early endosomes, triggering their dissociation. Subsequently, the second outer capsid protein, VP5, undergoes major changes in late endosomes, forming a membrane-penetrating pore that releases the transcriptionally active inner core into the host cytoplasm. Core assembly also proceeds stepwise and requires the accurate packaging of 10 positive-sense RNA segments. These segments form an RNA–RNA interaction network independent of viral proteins, beginning with the smaller segments and guiding the complete genome assortment. The small capsid protein, VP6, interacts with VP3 to facilitate RNA encapsidation. While infectious cores assemble in vitro without non-structural proteins, NS2 is essential for the in vivo formation of viral inclusion bodies via liquid–liquid phase separation, concentrating viral components and promoting genome assembly. These comprehensive characterizations of BTV provide a foundation for future control strategies against related reoviruses.

## 1. Introduction

Bluetongue virus (BTV) does not directly affect human health; however, it can be devastating to farmers’ livelihoods, as it not only causes high mortality in farm animals and reduces milk production, but also affects trade and disease control activities. Europe remained largely free of BTV until 2006, when the highly virulent serotype BTV-8 (one of the 28 known serotypes) suddenly emerged in several European countries for the first time, causing devastating consequences across southern, central, and northern Europe [1]. These outbreaks were largely associated with rising temperatures that created favourable conditions for *Culicoides* vectors—the natural transmitters of BTV—as well as with increased animal population density and movement.

How rising temperatures are influencing vector populations and what measures can be taken to safeguard livestock have become major concerns among farmers and veterinary professionals. This concern has intensified due to the recent emergence of a new serotype, BTV-3, at higher latitudes in Europe, which is currently spreading across several European countries [2].

Although the emergence of BTV in Europe is relatively recent, the virus has been endemic in many regions of the world for several decades, and it is recognized as an agent of economically significant veterinary disease. Over the past three decades, our focus has been on BTV, not only because of its impact on animal health, but also because it shares key biological and structural features with several scientifically and medically important viruses, including reoviruses and rotaviruses.

Our research has been dedicated to fundamental studies of the molecular and structural biology of BTV and its replication cycle. A detailed understanding of the different stages of the viral life cycle is essential for the development of improved strategies for the diagnosis, control, and prevention of viral diseases affecting both animals and humans. Multidisciplinary approaches have enabled significant advances in our understanding of BTV replication, including virion structure, mechanisms of host–cell entry, virus assembly, egress, and cell-to-cell transmission.

As a result of these efforts, BTV has become one of the most comprehensively characterised viruses at the molecular level. Given the extensive body of existing literature already available (see reviews: [3,4,5,6,7,8]), this article focuses on selected recent advances from our group that has enhanced our understanding of BTV entry and assembly mechanisms.

## 2. BTV Outer Capsid and Virus Entry Mechanisms

**Figure 1:**
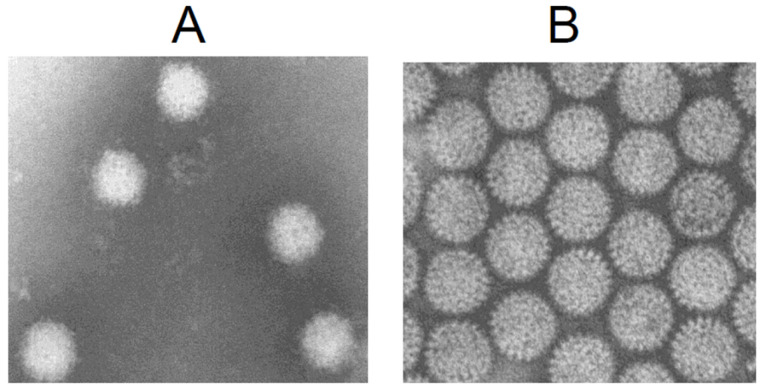
Negatively stained BTV virions (**A**) and core particles (**B**), showing that whole particles are not well defined, while core particles are not distorted.

Unlike most insect-borne viruses, the BTV particle lacks a lipid envelope, and negatively stained virus particles observed under the electron microscope appear as distorted spherical structures [9]. The viral particle is composed of two capsids: an outer capsid consisting of VP2 and VP5 and an inner capsid which, unlike the outer capsid, exhibits a distinct morphology with well-defined capsomers (Figure 1). The inner capsid, termed the core, consists of two concentric protein layers composed of VP7 and VP3, along with the transcription complex made up of three minor proteins (VP1, VP4, and VP6) [5,10]. The BTV genome comprises 10 double-stranded RNA (dsRNA) segments (S1 to S10). In infected cells, three or four additional non-structural proteins are synthesised to facilitate viral replication within the host cell [11,12].

**Figure 2:**
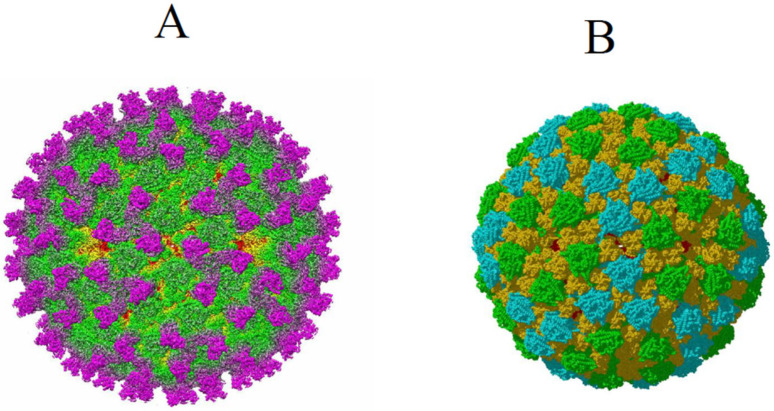
**Cryo-EM reconstruction of the BTV virion at 3.5 Å resolution**. (**A**) Cryo-EM density map of the BTV virion shown as radially coloured surface representation. Radially coloured surface representation (VP2 trimers: magenta, VP5: green, VP7: yellow, and VP3: red). (**B**) Cryo-EM density map of the BTV virion lacking VP2, shown as radially coloured surface representation of VP5 trimers (green and Cyan) and VP7 trimers (yellow) [15,16].

A series of three-dimensional structural studies using cryo-electron microscopy (cryo-EM) have revealed that BTV particles contain two architecturally complex capsids. The two outer capsid proteins, VP2 and VP5, which are involved in virus entry, possess very distinct structures, indicating distinct functional roles. VP2 is arranged as 60 trimeric spike-like structures protruding from the surface of the virion, whereas VP5 is arranged as 120 trimers that are more globular in shape and less exposed on the virion surface (Figure 2) [13,14,15,16,17].

VP2 is responsible for the haemagglutination activity and initiates cell attachment by binding to α2,3- and α2,6-linked sialic acids in a cell-type-dependent manner [18]. It is the most variable of the BTV proteins, and it is the primary determinant of virus serotype, bearing the majority of epitopes targeted by neutralizing antibodies. At least 28 BTV serotypes have been identified to date based on unique VP2 neutralisation profiles. However, low-level cross-neutralisation between certain heterologous serotypes has been observed in vaccine trials [19]. In addition, different serotypes share common VP2 epitopes that are either non-neutralizing for both serotypes and non-neutralizing in the conformational context of the heterologous VP2 protein [20].

**Figure 3:**
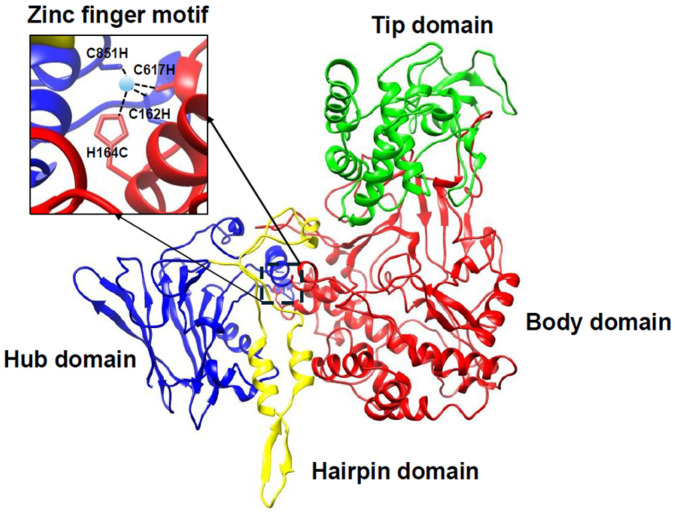
**Cryo-EM density map of VP2 monomer.** Four distinct domains designated as hub (blue), hairpin (yellow), body (red), and tip (green). A zinc-finger motif (CCCH tetrahedron) located at the junction of hub and body domains shown as amplified inset [16].

The atomic structure of the surface protein, VP2, from BTV-1 revealed four structural domains in each monomer: the hub, hairpin, body, and tip [15,16] (Figure 3). The hub domains of three monomers associate to form triskelion-shaped spike trimers that project from the outer capsid. At the interface between the hub and body domains, there is a well-coordinated zinc-finger motif responsible for conformational changes during cell entry.

The tip domain, located at the apex of the VP2 monomer, is prominently positioned for immune surveillance and antibody binding; a disruption of this region can inhibit viral attachment and entry into host cells.

**Figure 4:**
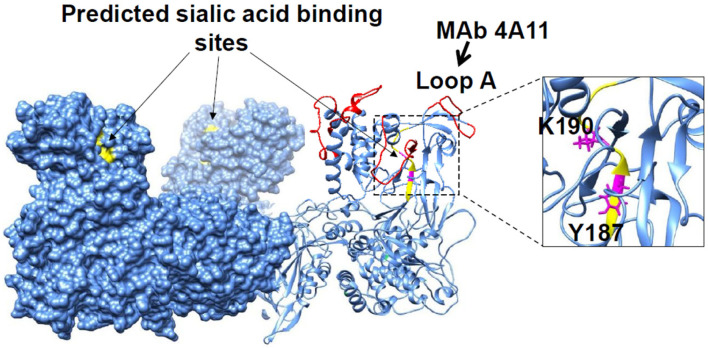
**Potential sialic acid binding sites (yellow) in VP2 identified by HDX-MS.** VP2 triskelion formed by three VP2 monomers showing the protruding three tip domains. Conserved amino acid residues, Y187 and K190, within peptide, VAYTLKPTYD (185aa–194aa), located at the tip domains of VP2 trimer are predicted to bind sialic acid. Four large flexible loop regions A–D (red) of the tip domain contain residues critical for neutralizing antibodies. BTV-1 serotype-specific neutralizing MAb 4A11 targets, specifically epitopes, within loop A [15,21,22].

In a recent study, the structural basis of serotype specificity was investigated through in silico structural comparisons between VP2 from BTV-1 and BTV-8, a highly virulent serotype. The resulting data were used to generate targeted recombinant VP2 mutants and mutant viruses via reverse genetics. An analysis using a panel of monoclonal antibodies, mutant VP2 proteins, and mutant viruses demonstrated that structural differences in the tip domain between these two distant serotypes constitute the primary targets of neutralizing antibodies. Furthermore, a mutation at a specific monoclonal antibody–binding loop region (Loop A; e.g., mAb 4A11) led to the loss of neutralizing antibody recognition (Figure 4). These findings also demonstrate the impact of VP2 structure on antigenic variation and differential virulence during outbreaks [21].

**Figure 5:**
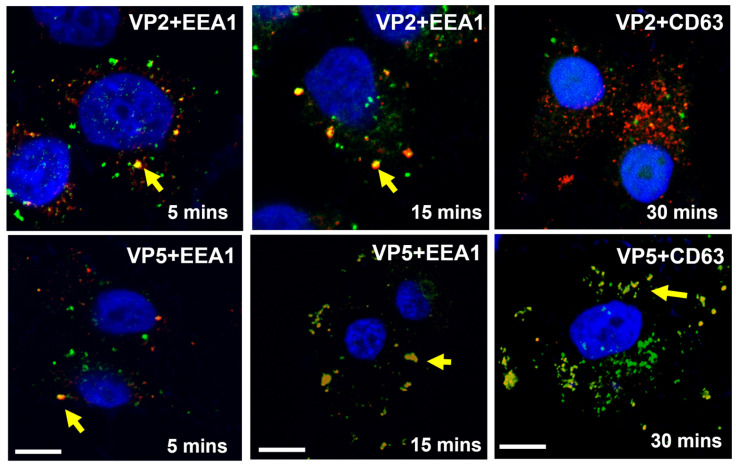
**Entry of BTV in early and late endosomes.** BTV bearing a tetracysteine-tagged VP2 in permeabilized cells VP2 (green) remains in early endosome but does not move to late endosome. The remaining particle, including VP5 (red), moves to late endosome. Arrows indicate colocalizations of VP2 and VP5 with endosomal markers [23].

Following the attachment, BTV enters host cells predominantly via a clathrin-mediated endocytic pathway, indicating that an acidic pH is required for viral entry. BTV particles are rapidly internalized after adsorption. VP2 is shed from the virion in early endosomes, while the remaining particles, including VP5, are trafficked to late endosomes (Figure 5) [23]. A single histidine residue that forms part of a tetrahedral zinc-finger motif (CCCH) in VP2 appears to be responsible for sensing the mildly acidic pH (6.0–6.5) of the early endosome and for triggering VP2 dissociation from the particle [16,24]. Both the conformation of the zinc-finger motif and the correct positioning of this histidine residue are critical for this function [22].

**Figure 6:**
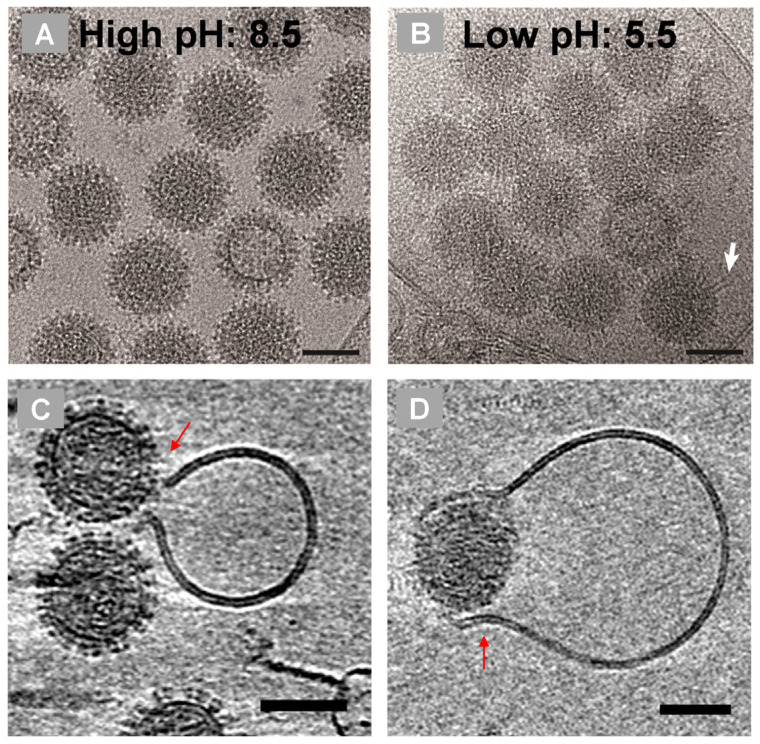
**Morphology of virus particles changes at low pH.** Cryo-EM images of BTV at high pH (**A**) and at low pH (**B**), showing that VP2 detached entirely; VP5 conformation changes substantially to an extended filament structure (**C**,**D**). The interaction of BTV with liposomal membrane and slices of cryo-ET reconstruction of the pH shift condition, showing membrane pore expansion and accommodated virus passage (arrow) [26].

**Figure 7:**
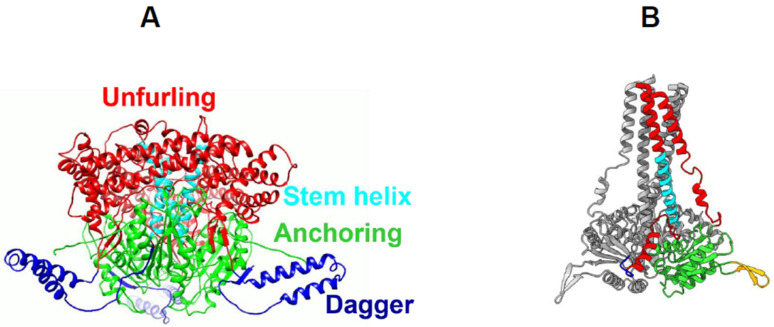
**Structure of BTV VP5 trimers in two different conformers.** (**A**) Side-view of a VP5 trimer showing three distinct domains: dagger, unfurling, and anchoring. (**B**) Structure of VP5 trimer in low pH state, with one monomer coloured and the other two monomers in grey [16,26].

A series of molecular analyses, combined with cryo-EM and cryo-tomography (cryo-ET) studies, has revealed how VP5 becomes functionally activated in the late endosome, where it senses the lower pH (5.0–5.5) and facilitates the transfer of the intact inner core into the host cell cytoplasm (Figure 6) [24,25,26]. At neutral to high pH (7.5–8.0), each VP5 monomer exhibits three distinct domains: the dagger, unfurling, and anchoring domains. Each trimer is organised around a stem–helix bundle, characteristic of fusion proteins from enveloped viruses, and contains clusters of histidine residues distributed across the unfurling and anchoring domains (Figure 7).

At late endosomal pH (6.0–5.5), the VP5 trimer undergoes a major conformational rearrangement, transforming into an elongated stalk through stepwise refolding of the unfurling domains into a six-helix bundle that protrudes from a triangular base, exposing the previously hidden dagger domain (Figure 7B). Simultaneously, a surface loop within the anchoring domain refolds into a hairpin structure, anchoring VP5 to the VP7 layer of the core. These extensive conformational changes are histidine-dependent, as single substitutions of selected histidine residues prevent the recovery of infectious virus. The stalk and dagger domains, together with a membrane-binding motif (WHXL) located within the anchoring domain, form a single pore in the endosomal membrane, enabling core release into the cytosol. Concurrently, the buried surface area of the VP5 trimer decreases substantially, facilitating its detachment from the core [26,27].

In summary, VP2 mediates the initial attachment to mammalian host cells, whereas VP5 is responsible for pore formation in the endosomal membrane, allowing core entry into the host cytoplasm. This stepwise and coordinated entry mechanism of BTV relies on precise sensing of pH changes at successive stages of endocytic trafficking.

## 3. Replication and Virus Assembly

The final product of BTV disassembly is a transcriptionally active, double-layered particle capable of transcribing genomic RNAs. This transcriptionally active core can be readily extracted from the virion using detergent treatment, which not only facilitates functional studies but also enables structural analysis at the atomic level. The surface layer of the core particle is composed of 260 VP7 trimers that coat an internal layer formed by 60 VP3 dimers [28]. Enclosed within the core is the viral transcriptase/polymerase complex—consisting of VP1 (RNA-dependent RNA polymerase), VP4 (capping enzyme), and VP6 (RNA-binding and packaging protein)—together with the 10 genomic dsRNA segments (S1 to S10) (see review [5]).

The atomic structures of each of the five core proteins have been resolved, and their structure–function relationships have been extensively described in the literature [5,29]; therefore, they will not be discussed in detail here. Within the core, the 10 genomic dsRNA segments are repeatedly transcribed by the polymerase complex to generate mRNAs for viral protein synthesis. Later, they also serve as templates for negative-strand RNAs for progeny genome synthesis. Notably, recombinant VP1 alone is capable of performing the synthesis of genomic RNA segments in vitro using either positive- or negative-sense RNA templates [30,31].

**Figure 8:**
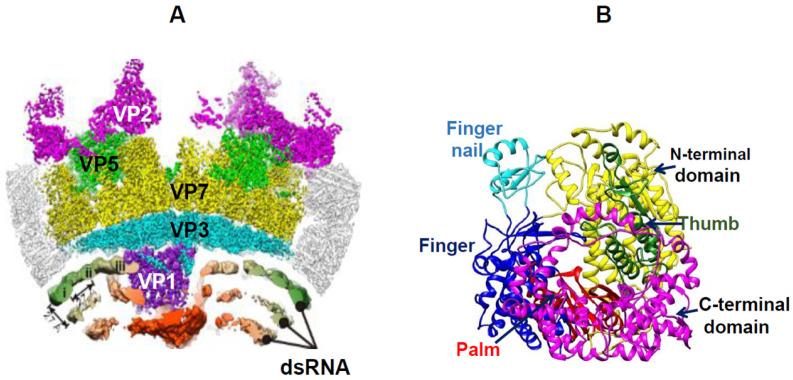
**Cryo-EM reconstruction of the vertex region of the BTV virion.** (**A**) The cryo-EM density map of BTV vertex on its side-view. Protein densities are coloured as follows: VP2, magenta; VP5, green; VP7, yellow; VP3, cyan; and VP1 polymerase, purple. RNA densities are low-pass-filtered and radially coloured. (**B**). Ribbon model of VP1 polymerase in quiescent state showing different domains as indicated. Polymerase domain shows the extra fingernail motif (cyan) on the top of BTV finger subdomain (blue) [32].

VP1 polymerase is anchored to the inner surface of the capsid shell through the five asymmetrically arranged N-terminal regions of the surrounding VP3 molecules (Figure 8B) [32]. However, the removal of VP2 and VP5 during entry triggers both large-scale rearrangements of the capsid shell and localized structural changes in interacting VP3 molecules, thereby priming VP1 within the capsid for transcription. VP1 possesses two unique domains in addition to the conserved “hand-shaped” polymerase core: an N-terminal domain that opens the genomic RNA duplex to isolate the negative-strand template, and a C-terminal domain that separates the emerging template–transcript duplex, guiding genome reannealing and forming a transcription bubble (Figure 8B) [32]. The VP3 shell transiently opens to extrude newly synthesised transcripts. These two additional VP1 domains interact with an N-terminal latch of the inner capsid protein that regulates polymerase activity. In pre-entry virions, the latch is engaged, whereas in the post-entry transcriptionally active state, it becomes disengaged [33]. Each newly synthesised RNA transcript with a 5′-capped structure exits the core through a distinct channel rather than randomly, as was previously believed [34].

To elucidate the genome replication process of BTV, two innovative systems were developed: a reverse genetics (RG) system and a cell-free assembly (CFA) system [35,36]. Together, these tools have significantly advanced our understanding of the BTV life cycle.

Using the RG system, we showed that the transfection of 10 single-stranded positive-sense RNA transcripts (+ssRNAs) into mammalian cells is sufficient to establish BTV infection. This approach allowed the identification of the components of the primary replicase complex, revealing that, in addition to the +ssRNAs encoding the inner capsid proteins, the non-structural protein, NS2, is essential for initiating viral replication [37].

In contrast, the CFA system enables the in vitro assembly of infectious BTV core particles without requiring NS2. In this assay, the 10 +ssRNAs first associate with the three polymerase complex proteins, followed by the encapsidation with VP3 to form a subcore particle and subsequent addition of VP7, to produce a stable core. When all four nucleoside triphosphates are included prior to VP7 incorporation, the packaged +ssRNAs are converted into genomic dsRNAs. These reconstituted core particles are capable of initiating BTV replication in susceptible host cells [37].

Furthermore, the CFA assay enabled the determination of the sequential recruitment of 10 +ssRNA segments into the viral capsid, as discussed in the following section.

## 4. Genome Segment Assortments and RNA Complex Formation

RNA packaging of BTV is challenging because it must not only selectively incorporate ten ssRNAs but also ensure that at least one complete set of all 10 segments is accommodated within the limited space of the viral core. The mechanisms by which BTV +ssRNAs are recognized and packaged with precise stoichiometry have been studied in detail [38,39,40]. The 10 BTV genomic segments vary in length; however, both termini of each segment contain highly conserved complementary sequences of five or six nucleotides. Although these conserved sequences are present in all segments, the length of the 3′ untranslated region (3′ UTR) differs among the 10 segments [41].

**Figure 9:**
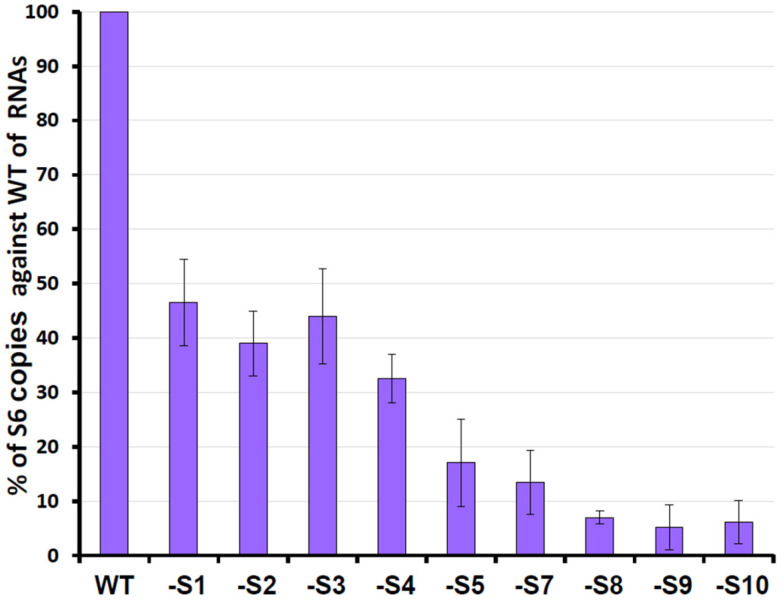
**Exclusion of specific BTV RNA segment influences genome packaging**. A total of 10 BTV ssRNAs (WT), along with ssRNAs that lacking one ssRNA each at a time (-S1, -S2, etc.) were used in the CFA assay. The packaged ssRNA in the relevant core-containing fraction was purified and quantified through qRT-PCR to determine the packaging efficiency. The efficiencies are shown in percentages, and standard deviations (error bars) were calculated [38].

Secondary structure analysis using RNAfold predicted interactions between the complementary 5′ and 3′ hexanucleotides, forming hairpin and stem–loop structures separated by a potentially flexible stretch of nucleotides. The deletion of either the 5′ UTR or the 3′ UTR, with or without the conserved regions, or the introduction of mutations within the UTRs that altered RNA structural conformation, prevented RNA packaging [42]. Furthermore, by excluding one genomic segment at a time, it was possible to show that BTV genome segments are not packaged individually but rather through interacting networks among different segments, with network formation most likely initiating with the smaller segments (Figure 9) [38,43].

**Figure 10:**
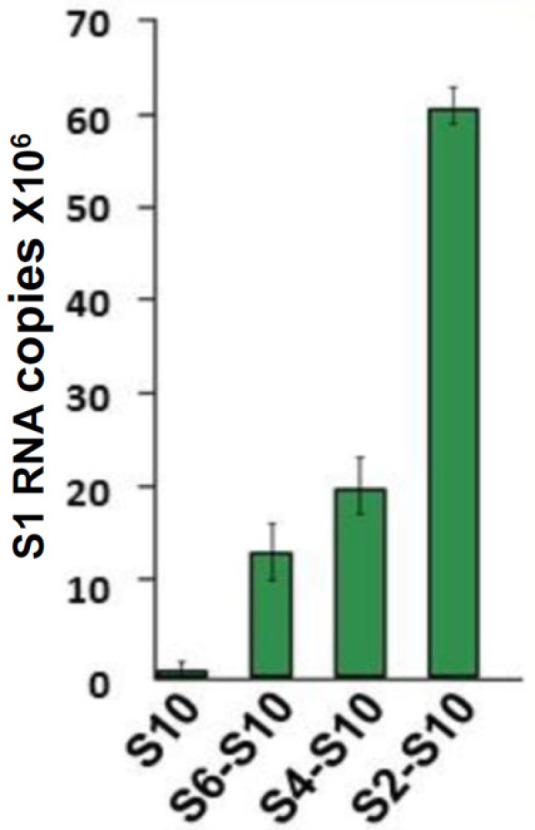
RNA–RNA interactions and genome assembly proceed sequentially from smaller to larger segments. BTV S1 was incubated with S10-coated beads either alone or with mixtures of other segments. The highest number of S1 RNA copies was pulled down in the presence of S2–S10, indicating that S10 interacts with other segments sequentially, from smaller to medium and ultimately to the largest segments [38].

Subsequent studies demonstrated that the secondary structure of smallest S10 is critical for packaging. An in vitro biotinylated primer-coated streptavidin bead assay revealed that S10 has a high affinity for three smaller ssRNA segments (S7–S9) but not for the larger segments. Pull-down experiments using different combinations of ssRNA segments further suggested that RNA–RNA interactions occur in a defined order necessary for RNA complex formation. This process initiates with the smaller segments, which first assemble into an RNA complex that subsequently interacts sequentially with additional segments (S6, S5, S4, etc.), with S1 being the final segment to associate with the complex (Figure 10).

Distinct RNA complexes comprising at least the five smaller segments (S6–S10) were visualised through the electrophoretic mobility shift assay (EMSA). These complexes could be disrupted by short antisense oligoribonucleotides (ORNs) complementary to the 3′ UTR of S10. The same ORNs also inhibited RNA packaging in a cell-free assembly (CFA) assay and significantly reduced virus replication in cell culture [44]. Together, these findings indicate that RNA–RNA interactions are essential for triggering BTV genome segment packaging into the assembling capsid and that specific RNA sequences mediate these interactions. Trans-interactions between the segments at multiple defined sites were subsequently identified using bespoke bioinformatic predictions and were validated through targeted mutagenesis and reverse genetics analyses [43,44].

**Figure 11:**
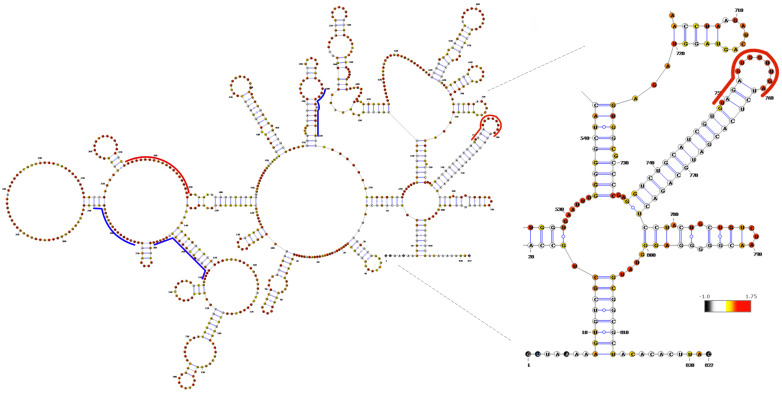
**Secondary structure with integrated SHAPE data of BTV S10**. The secondary structure of S10 was generated through RNAfold. SHAPE reactivities for each nucleotide position are show in the bar using a continuous colour scale (−1 black, 0 white, 0.3 yellow, 0.7 orange, and 1+ red). Red (increase) and blue (decrease) lines on the uncomplexed S10 indicate the regions that changed during the complex formation [39].

**Figure 12:**
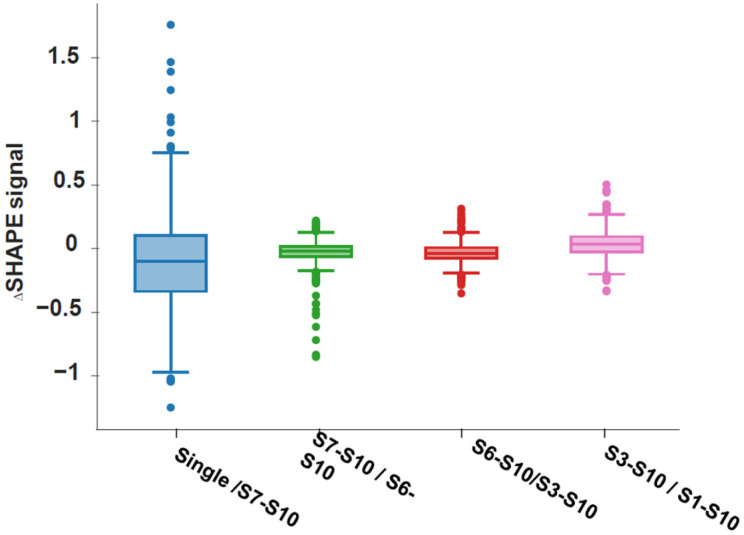
**The SHAPE profiles of S7–S10 in different stages of RNA complex.** Boxplots show the distribution of values obtained in different complexes, S7−S10, in singlet form and in different complexes. A wider distribution indicates more extreme values between the two complexes compared [39].

The concept of multipartite genome segment selection via an RNA interaction network prior to or during packaging is attractive, as it eliminates the need for the assembling virus to independently recognize multiple RNA molecules. To unequivocally establish this concept and to obtain direct experimental evidence for the points of contact among interacting segments, we determined the structure of each BTV transcript, both individually and in different combinations, using 2′-hydroxyl acylation analyzed by primer extension and mutational profiling (SHAPE-MaP). The SHAPE-MaP analysis identified RNA structural changes associated with complex formation and revealed putative RNA–RNA interaction sites (Figure 11). These data further demonstrated the existence of a core RNA complex, composed of four smaller segments (S7–S10), that serves as an “anchor” for the sequential assembly of a complete network containing all 10 ssRNA segments (Figure 12) [39].

**Figure 13:**
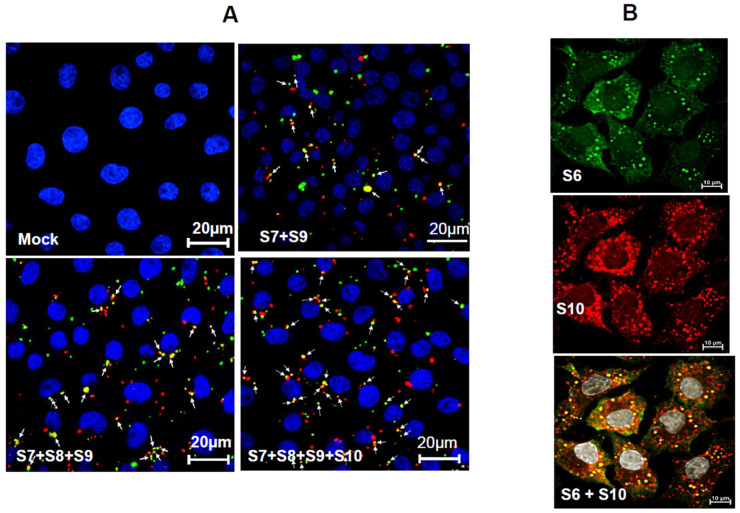
**Viral RNAs fluorescence in situ hybridization chain reaction (HCR) and colocalization analyses.** (**A**) BSR cells transfected with no RNA or BTV ssRNAs (S7 + S9, or S7 + S8 + S9, or S7 + S8 + S9 + S10). Transfected cells were fixed and in situ HCR was carried out using multiple hairpin nucleotide probes. Two colour fluorophores conjugated to the hairpin nucleotide probes were used to detect S7 and S9 BTV RNA segments in the complexes. (**B**) BSR cells were infected with BTV, fixed and incubated with probes targeting S6 and S10 ssRNA segments for HCR and colocalization analysis. Colocalization (arrows) analysis revealed yellow spots, indicating the overlap of Cy3 and Cy5 signals of S6 and S10 in the RNA complexes [45].

The same hierarchical order of core RNA complex formation was observed in cells transfected with viral RNAs [35]. Notably, no viral proteins were required for these RNA–RNA interactions or for complex formation. A combination of nucleotide substitution analysis, in vitro RNA packaging assays (CFA), and the in vivo BTV reverse genetics (RG) system identified critical RNA structures within core segments that are required for the generation of infectious BTV. More recently, using a novel RNA fluorescence in situ hybridization chain reaction (HCR) approach combined with colocalization analysis, the dynamic nature of BTV +ssRNA interactions within infected cells was demonstrated. These studies confirmed that viral RNA segments interact with each other to form a 10-segment RNA complex in vivo, consistent with observations from in vitro studies, and that this process is independent of BTV proteins (Figure 13) [45].

## 5. RNA Packaging Mechanisms

The assembly of empty virus-like particles (VLPs) composed of four major proteins—VP2, VP5, VP7, and VP3—or core-like particles (CLPs) of VP3 and VP7 via recombinant protein expression systems is highly efficient, yielding stable structures [46,47,48]. Empty CLPs can also incorporate VP1 polymerase and the mRNA capping enzyme, VP4, either independently or as a VP1–VP4 complex [10,42]. In contrast, VP6 is not readily incorporated into CLPs. However, recombinant VP6 can interact efficiently with recombinant VP3 to form a heterohexameric complex consisting of two copies of VP6 and four copies of VP3 [29].

**Figure 14:**
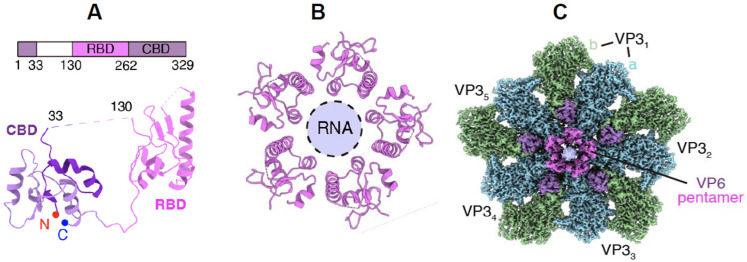
**Three-dimensional atomic structures of VP6 of BTV.** (**A**) Domain organisation and atomic model of VP6 showing RNA-binding domain (RBD) and capsid-binding domain (CBD). (**B**) Pentameric configuration of VP6 and RNA-binding tunnel. (**C**). Cryo-electron microscopy (cryo-EM) density of VP3, VP6, and RNA in the sub-particle reconstruction from pre-subcore. The RNA density at the 5-fold axis shown in light purple (modified from [29]).

BTV VP6 (36 kDa), the smallest capsid protein, has been identified as a crucial component of the primary replicase complex [37]. In the absence of functional VP6, genomic RNA segments are not packaged; however, VP6-deficient virus remains infectious in VP6 complementary cells [49,50]. VP6 is a highly positively charged protein with strong RNA-binding affinity, and it possesses nucleoside triphosphatase activity [51]. It plays a vital role in the early stages of capsid assembly through its interaction with VP3, which is essential for RNA packaging [52,53]. Recent high-resolution structural analyses have revealed that VP6 is located as a pentamer beneath the vertices of the VP3 shell, at the C-terminal regions. Each VP6 molecule consists of two domains: the capsid-binding domain (CBD), formed by the N- and C-terminal regions, and the RNA-binding domain (RBD), formed by the central region (Figure 14) [29]. Structural data further revealed that the interactions between VP3 and VP6 create a tunnel at the 5-fold axis, facilitating RNA packaging and subsequent expansion of the VP3 shell, thereby contributing to subcore stability. It is likely that newly synthesised ssRNA complexes are packaged during subcore assembly, with VP6 simultaneously binding to ssRNAs. VP6 pentamers form the tunnel together with VP3 during this process. As demonstrated by CFA assays, RNA replication occurs after subcore assembly or during core formation. Notably, to date, structural studies have failed to identify VP6 in mature cores or virions, suggesting flexibility in its arrangement or that VP6 may be obscured by genomic RNA within the subcore and core.

## 6. Non-Structural Protein NS2 and Core Assembly

**Figure 15:**
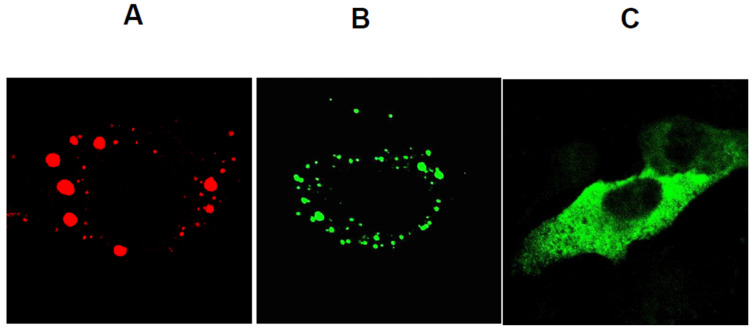
**The formation of inclusion bodies by NS2 is impaired when NS2 is not phosphorylated**. (**A**) BHK cells were infected with BTV and NS2 (VIBs), which was visualised using a TRITC-conjugated antibody (red). (**B**,**C**) BHK-21 cells were transfected with plasmids encoding NS2 or NS2 mutant lacking the phosphorylation site. Expressed recombinant NS2 and NS2 mutants were visualised using an FITC-conjugated antibody (green) [55].

Although NS2 is not required for the assembly of recombinant CLPs or VLPs lacking genomic RNA, nor for the in vitro assembly of infectious core particles (as indicated by CFA assays), it is an essential component of the primary replicase complex, responsible for initiating secondary replication in infected cells. NS2 is a phosphorylated protein and the principal constituent of membraneless, globular structures in the cytoplasm of infected cells (Figure 15), known as viral inclusion bodies (VIBs) [54,55,56]. During BTV replication, NS2 associates with newly synthesised core proteins and viral ssRNA transcripts [57]. Phosphorylation of NS2 is critical for VIB formation, which serves as the cytoplasmic site of core assembly [55].

Non-phosphorylated recombinant NS2 can still interact with BTV proteins and ssRNA segments. Moreover, a recent comprehensive study using RNA fluorescence in situ HCR assay, combined with site-specific mutagenesis and reverse genetics, revealed that RNA–RNA interactions and RNA complex formation in BTV-infected cells occur sequentially from the smallest to largest RNA segments and are independent of NS2/VIBs. However, NS2 appears to facilitate or enhance the assembly of larger RNA segments in infected cells. The study also indicated that the smallest core protein, VP6, associates with the RNA network prior to VIB formation and is recruited to VIBs only in the presence of viral RNAs—not cellular RNAs—implying a selective mechanism favouring viral RNAs. VP6 may also function as a checkpoint, regulating RNA complex formation and preventing potential cytotoxicity [45]. Although VIBs and NS2 are not directly involved in RNA–RNA interactions, they play a crucial role in efficient viral genome assembly—especially for larger RNA segments—while being dispensable for the initial formation of RNA complexes.

**Figure 16:**
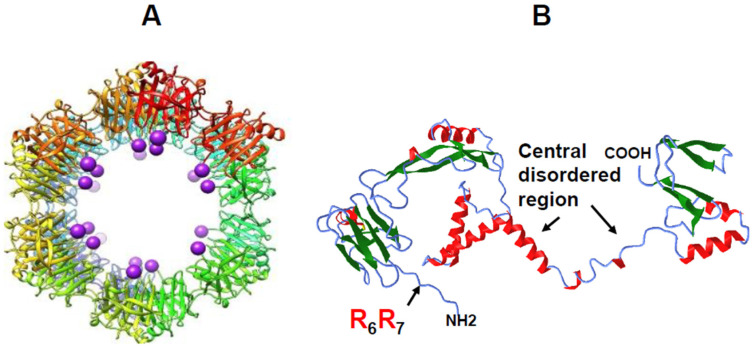
**Cryo-electron microscopy of NS2 oligomer showing the ribbon and surface representations of oligomeric NS2 N-terminal domains**. (**A**) The model is generated based on the crystal structure of NS2 N-terminal domain, and the C-terminal ends of each NS2 N-terminal domain are shown in purple spheres. (**B**) A model of full-length NS2 predicted by Rosetta showing an extended molecule with β-sheet-rich domains (green) in the both N- and C-termini connected by a central disordered region consisting of three α-helices (red) and coil (blue). BTV ssRNA binding site at arginine residues is indicated. The model suggests a molecule that can undergo significant secondary structural changes [60,61].

In addition to viral components, NS2 interacts with host proteins such as Casein Kinase 2 (CK2), Phosphatase 2A (PP2A), and Ca^2+^, with each interaction being essential for viral replication [58,59,60]. Non-phosphorylated NS2 oligomerises into a decamer, forming a cage-like structure with a central Ca^2+^-binding region (Figure 16) [60]. Current evidence suggests that NS2 coordinates VIB assembly and disassembly via phosphorylation by CK2 and dephosphorylation by PP2A, while Ca^2+^ binding induces conformational changes that expose key phosphorylation sites. This regulated assembly and disassembly process is critical for viral replication and for the release of newly assembled cores from VIBs during virus maturation.

**Figure 17:**
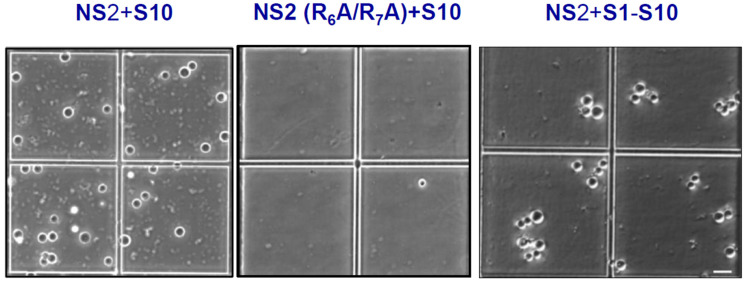
**Visualization of NS2 + RNA condensates under phase contrast microscope.** Phase separation of recombinant NS2 or mutant NS2 (R_6A_ + R_7A_) incubated with S10 showing NS2 lacking RNA-binding site did not form condensates when NS2 with 10 RNA segments enhanced condensate formation [61].

Recent studies have further elucidated how NS2 and VIBs coordinate multicomponent core assembly and release [61]. Viral assembly factories, such as VIBs, can exist in different liquid states—ranging from condensed to dilute—through the process of liquid–liquid phase separation (LLPS) in the cytoplasm. LLPS is driven by multivalent interactions, often involving phosphorylation and RNA binding, and it is frequently mediated by intrinsically disordered regions (IDRs). NS2, the only phosphorylated RNA-binding protein of BTV, contains β-sheet-rich domains at both the N-terminal (aa 1–169) and C-terminal (aa 266–354) regions, connected by a central disordered region (Figure 16B). Recent biophysical, biochemical, and biological analyses have revealed that NS2 undergoes LLPS to form both dilute and condensed phases (Figure 17). Its phosphorylation state and RNA-binding affinity regulate these phase separations, which are accompanied by changes in secondary structure.

**Figure 18:**
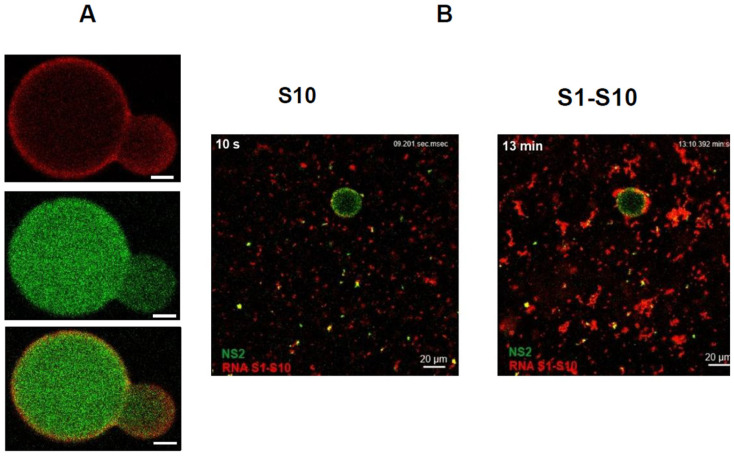
**Visualization of NS2 + RNA condensates as large droplets under phase contrast microscope.** (**A**) BTV S10 RNA (1 mg/mL) stained with GelRed was observed colocalising with NS2 stained with green fluorescence in phase-separated condensates, visible as large droplets after 40 min of incubation. (**B**) Visualization of preferential phase separation of the RNA–RNA complex (S1-S10) over a single mRNA segment (S10) with NS2 [61].

Moreover, specific arginine–RNA interactions within phosphorylated NS2 drive LLPS, coordinating assembly dynamics (Figure 17). We also observed that when transient NS2–viral RNA condensates were incubated with a higher concentration of BTV RNA transcripts, many transcripts crossed the phase boundary at the condensate periphery and entered the condensates (Figure 18). Conversely, RNA-filled NS2 condensates responded to mild pH-induced conformational changes in NS2 by releasing transcripts from inside the condensates to the exterior, crossing the phase boundary without disrupting condensate assembly. Interestingly, when the RNA packaging protein, VP6, was added, most RNA molecules were retained within the condensates, likely facilitating packaging and subcore assembly.

## 7. Concluding Remarks

**Figure 19:**
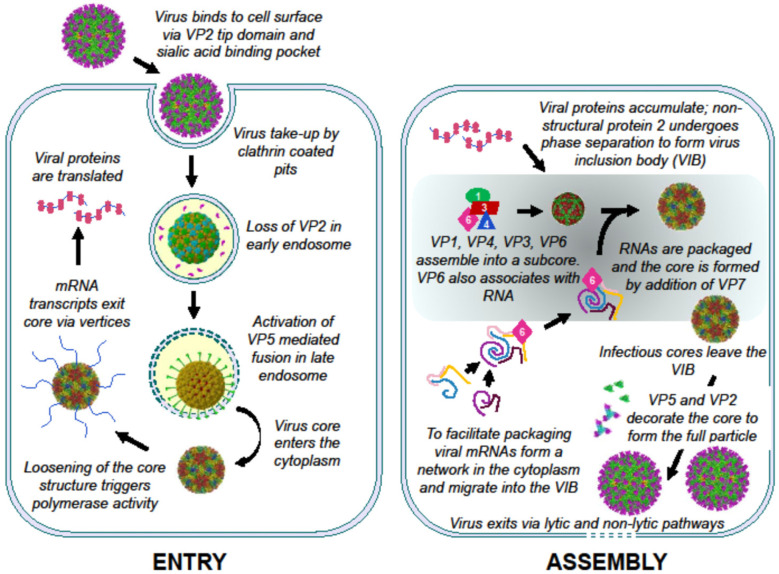
**Schematic diagrams of Virus Entry and Assembly**.

BTV remains a critical subject of study due to its significant impact on global agriculture and its role as a biological model for other complex double-stranded RNA viruses. Decades of multidisciplinary research have made BTV one of the most comprehensively characterized viruses at the molecular level. This review particularly highlights recent research on certain key steps of virus life cycle, as illustrated in the schematic diagram (Figure 19). In particular, our recent study has elucidated a sophisticated, stepwise entry mechanism where the outer capsid proteins, VP2 and VP5, act in a highly coordinated manner. VP2 initiates cell attachment and senses the mildly acidic environment of early endosomes to trigger its own dissociation, while VP5 subsequently undergoes dramatic conformational changes in late endosomes to facilitate core release into the host cytosol.

The assembly of the BTV genome is equally complex, governed by a hierarchical RNA–RNA interaction network. Crucially, this process of segment assortment and complex formation is independent of viral proteins, demonstrating that the RNA segments themselves contain the necessary information for their own recruitment. While the minor capsid protein, VP6, is essential for the physical packaging of these RNAs into the subcore, the non-structural protein, NS2, provides the necessary environment for efficient replication in vivo. Through liquid–liquid phase separation (LLPS), NS2 forms viral inclusion bodies (VIBs) that act as “condensers,” concentrating viral components and facilitating the assembly of larger genome segments that might otherwise be less efficient.

Ultimately, the integration of structural biology, reverse genetics, and cell-free assembly systems has provided a detailed roadmap of the BTV life cycle. These advances into viral entry, genome assortment, and the role of phase-separated compartments not only enhance our fundamental understanding of virology but also provide the essential foundation for the development of next-generation diagnostics, vaccines, and control strategies aimed at protecting livestock against emerging BTV serotypes in the context of a changing global climate.

## Data Availability

No new data were created or analysed in this study.
